# A33 DIAGNOSTIC YIELD AND QUALITY OF EUS-GUIDED LIVER BIOPSIES IN A 142-PATIENT COHORT - THE KELOWNA EXPERIENCE

**DOI:** 10.1093/jcag/gwae059.033

**Published:** 2025-02-10

**Authors:** F Jowhari, S Neong, K Berg

**Affiliations:** Medicine, The University of British Columbia Okanagan, Kelowna, BC, Canada; Medicine, The University of British Columbia Okanagan, Kelowna, BC, Canada; Medicine, The University of British Columbia Okanagan, Kelowna, BC, Canada

## Abstract

**Background:**

EUS-guided liver biopsy (EUS-LB) is a safe & effective technique, consistently yielding diagnostic accuracy with adequate tissue samples. However, most studies are limited by small sample sizes, leaving gaps in data. Despite growing international evidence, a comprehensive Canadian experience remains unreported. To address this, we present our institutional data on EUS-LB, emphasizing diagnostic yield, safety & procedural benefits.

**Aims:**

To evaluate the diagnostic yield, safety & procedural efficacy of EUS-guided liver biopsies in a large cohort of patients from a Canadian healthcare setting.

**Methods:**

This is a retrospective analysis of patients who underwent EUS-LB over a 4 year period. All procedures were performed with a linear echoendoscope using a 1-2 pass,selective-actuation,wet suction technique & a 19-G Franseen-tip needle using our own institutional protocol. All patients received either conscious or deep sedation. Specimen adequacy was assessed by a GI histopathologist evaluating key parameters including the number of complete portal tracts (CPT),length of longest intact core (LIC),total specimen length (TSL) & degree of fragmentation, using guideline specific cutoffs where applicable. Comprehensive demographic, clinical & procedural data were also collected for detailed analyses.

**Results:**

A total of 142 consecutive patients underwent EUS-LB at Kelowna General Hospital, British Columbia, from April 2020 to October 2024. The median age was 62 years (IQR 52–70), with 109 women (77%) and 33 men (23%). The median BMI was 27 kg/m^2^ (IQR 24–32). The diagnostic yield was 99.3%, with all but one specimen being diagnostic. Adequate histological specimens were obtained in 136 patients (95.7%), and a conclusive diagnosis was reached in 141 patients (99.3%). The most common diagnosis was metabolic dysfunction-associated steatohepatitis (MASH) in 42 patients (29%), followed by drug-induced liver injury (14.5 %), autoimmune hepatitis (13.1%), and primary biliary cholangitis (PBC) (10.3%). At the time of this submission, detailed histopathologic analyses were available for a subset of patients. The median total specimen length (TSL) was 4.4 cm (IQR 3.3–5.2), the median number of complete portal tracts (CPTs) was 13 (IQR 11.5–23.5), and the median longest intact core was 1.3 cm (IQR 1.1–1.7). No major adverse events were observed.

**Conclusions:**

Our study reinforces the evolving role of EUS-guided liver biopsy in liver diagnostics, demonstrating its safety, efficacy, and high diagnostic yield. This technique not only ensures procedural success but also enhances the autonomy of gastroenterologists, promoting more timely and integrated patient care. As the largest study of EUS-guided liver biopsies conducted in a Canadian healthcare setting, it addresses a critical gap in the literature and further validates the role of EUS-LB in modern hepatology practice.

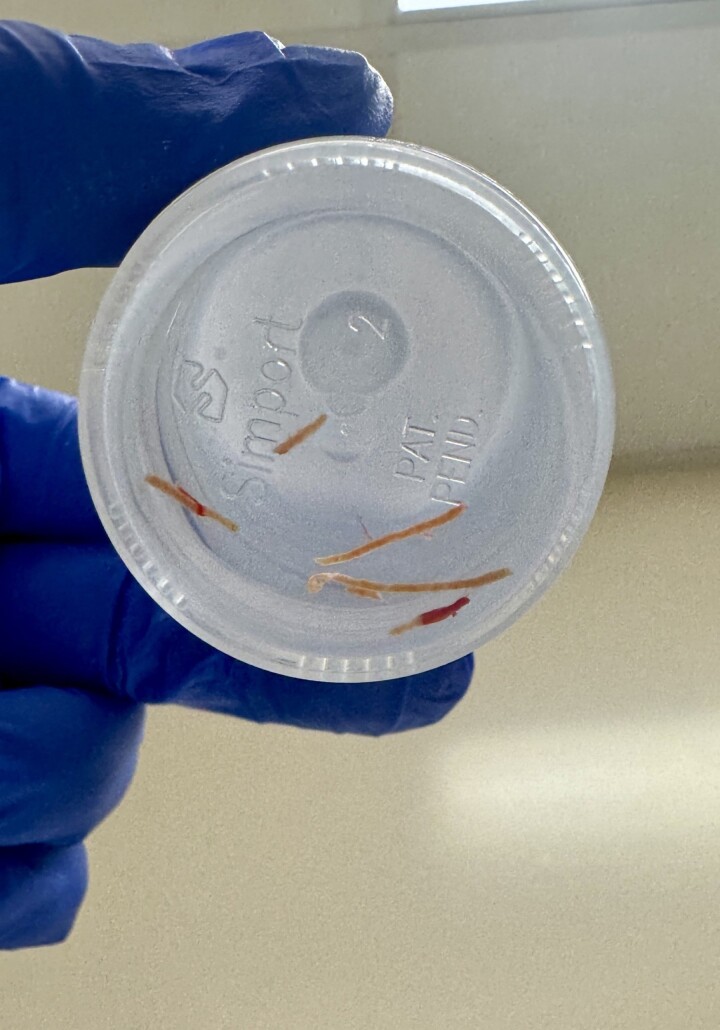

Gross intact core EUS-LB specimens collected in formalin.

**Funding Agencies:**

None

